# Drug Safety and Relevant Issues in the Real-World

**DOI:** 10.3390/ph16121689

**Published:** 2023-12-05

**Authors:** Maria Antonietta Barbieri, Natasha Irrera, Irma Convertino

**Affiliations:** 1 Department of Clinical and Experimental Medicine, University of Messina, 98125 Messina, Italy; nirrera@unime.it; 2 Unit of Pharmacology and Pharmacovigilance, Department of Clinical and Experimental Medicine, University of Pisa, 56126 Pisa, Italy; convertino.irma@gmail.com

The global impact of the COVID-19 pandemic has underscored the pivotal role of drug safety and effective communication within the realm of pharmacovigilance, particularly in times of unprecedented public health emergencies [1]. The advancements observed in clinical practice, elevating quality standards across all medical settings, can be attributed to the cumulative progress in medical knowledge and technologies over the years, with a growing reliance on real-world evidence. This evidence helps bridge the gaps present in clinical trials [2]. Randomized clinical trials, the gold standard for assessing drug efficacy and safety, face inherent constraints that became glaringly apparent during the pandemic. The restricted number of eligible participants and short follow-up periods often lead to an incomplete representation of the real-world population [3]. Notably, the use of drugs beyond the selected population in the pre-marketing phase allows for the effectiveness and safety profile of medications to be depicted in a continuous, evolving manner. Recognizing these limitations, the articles featured in this Special Issue emphasize the pivotal role of pharmacovigilance and pharmacoepidemiology studies. This approach implies that real-word studies not only contribute to the immediate assessment of the benefit–risk profile of new drugs post-approval but also offer insights into long-term effects, response to diseases, drug utilization patterns, and the occurrence of adverse events (AEs) for medications that have been on the market for extended periods [4,5].

The insights gained from these studies are not only crucial for ensuring patient safety but also for guiding evidence-based decision making in healthcare. Furthermore, the meticulous analysis of data sourced from healthcare administrative databases, spontaneous reporting systems for safety surveillance, clinical registries, and medical records [6] plays a pivotal role in tailoring therapeutic approaches to individual patients. Variables such as gender, age, disease severity, genetic factors, comorbidities, economic status, and polytherapy are considered, leading to the minimization of AEs and the optimal allocation of resources [7,8]. This patient-centric approach results in tangible advantages for both individuals and healthcare systems. In pursuit of advancing these principles, we are delighted to accept the invitation to be guest editors for this Special Issue of “*Pharmaceuticals*”. This volume presents review and original research papers by experts in areas relevant to the topic of “Drug Safety and Relevant Issues in the Real World”, with a focus on pharmacovigilance and pharmacoepidemiological studies. The objective is to offer valuable insights for physicians, pharmacists, and other healthcare professionals, making a meaningful contribution to the ongoing enhancement of the implementation of real-world evidence. This, in turn, fosters a more comprehensive and patient-focused approach to healthcare. A detailed list of these contributions is provided below.

A disproportionality analysis performed by Velișcu et al. (contribution 1) on data from the European Spontaneous Reporting System, EudraVigilance, after the safety concern issued by the Pharmacovigilance Risk Assessment Committee (PRAC) about the hepatobiliary disorders associated with cladribine, confirmed the risk of serious cladribine-induced hepatic adverse drug reactions (ADRs), primarily manifesting in adult females.

Socha et al. (contribution 2) conducted a study utilizing electronic medical records to compare the outcomes of labor induction between the vaginal administration of misoprostol and dinoprostone. The findings revealed that misoprostol was associated with a shorter time to delivery, decreased reliance on oxytocin, and a higher utilization of analgesia.

In a cross-sectional analysis of electronic medical records, Alwhaibi et al. (contribution 3) brought attention to the higher incidence of potentially inappropriate prescriptions among elderly women in Saudi Arabia than among elderly men. This gender disparity was not only limited to prescription patterns but also encompassed various clinical and socioeconomic aspects.

In their prospective study assessing treatments for immune thrombocytopenia in Egyptian patients, Hamed et al. (contribution 4) highlighted that corticosteroids exhibit an initial positive response as a first-line approach. However, it was noted that, over time, corticosteroids may be linked to disease worsening. In contrast, eltrombopag and romiplostim demonstrated a more favorable benefit–risk profile than other drugs in the study.

In a prospective analysis of patients undergoing proton pump inhibitor (PPI) treatment, Baiardi et al. (contribution 5) examined the implementation of a deprescription protocol. The study assessed clinical outcomes through a questionnaire, unveiling a commendable adherence among prescribers to the deprescribing strategy. Furthermore, the findings indicated minimal symptom relapse among the patients.

Galletti et al. (contribution 6) performed a prospective observational cohort study on patients treated with dupilumab for chronic rhinosinusitis with nasal polyps. They observed an optimal response to the treatment along with a low incidence of AEs at both the 6-month and 12-month follow-up periods.

In their population-based study, Gutiérrez-Abejón et al. (contribution 7) analyzed drug-dispensing data to assess the utilization of driving-impairing medicines. The study revealed a noteworthy increase in the use of these medicines over the years in Spain, emphasizing the necessity of integrating electronic prescription tools with targeted educational programs. Such integration aims to support prescribers, pharmacists, and patients in making informed decisions regarding the use of medications that may impact driving ability.

Convertino et al. (contribution 8) evaluated the first generation of Janus kinase inhibitors in the first 6 months after approval through a retrospective cohort study on Tuscan administrative healthcare databases and highlighted the use of these drugs in compliance with clinical guidelines and a selective prescription causing an increase in healthcare costs.

In their real-world analysis, Cortinovis et al. (contribution 9) examined data collected from administrative databases linked to pathological anatomy records. The study revealed that approximately half of early-stage non-small-cell lung carcinoma patients experienced a relapse, leading to healthcare costs for the Italian system that were nearly twice as high as those for patients who did not experience a relapse.

In their narrative review, Bellanca et al. (contribution 10) concentrated on elucidating the risk factors, pharmacogenetics/genomics, and management of ADRs in older adults. This emphasis stemmed from the substantial impact of these factors on morbidity and mortality, contributing significantly to the heightened economic burden on healthcare systems.

Syed et al. (contribution 11) delved into the drug safety profiles of medications prescribed for chronic pain, paying special attention to the potential risks of suicidality and suicidal ideation. The study was prompted by growing apprehension surrounding the escalating usage of both established and recently approved drugs over the years.

In their scoping review, Ciccone et al. (contribution 12) highlighted safety concerns associated with antiangiogenic drugs. The review identified gaps related to cardiovascular AEs that were neither reported in drug labels nor assessed in pharmacovigilance studies. Additionally, the study raised a signal regarding pericardial disease, indicating the need for further research to confirm this potential association.

Hung et al. (contribution 13) performed a systematic review and meta-analysis of cohort studies assessing the prolonged use of steroids preceding orthopedic surgery. The analysis focused on postoperative adverse events associated with this exposure, revealing a heightened risk of reported adverse events.

In conclusion, the relevant issues highlighted in this collection of real-world studies underscore crucial areas of concern and offer specific insights. First, this Special Issue involves addressing unmet clinical needs through the continuous monitoring of specific drugs, implementing surveillance programs for chronic medications, conducting investigations into long-term effects, and optimizing therapies in the early stages of diseases. Moreover, this Special Issue revolves around the adoption of specific therapeutic protocols for improved patient care, the implementation of deprescribing programs, the adoption of a multidisciplinary approach in therapy, and the integration of electronic tools to guide prescribing practices, promoting both appropriate prescriptions and encouraging patients’ adherence to treatment.
